# Pancreatic adenocarcinoma up-regulated factor (PAUF) enhances the accumulation and functional activity of myeloid-derived suppressor cells (MDSCs) in pancreatic cancer

**DOI:** 10.18632/oncotarget.10123

**Published:** 2016-06-17

**Authors:** Jinhoi Song, Jaemin Lee, Jinsil Kim, Seongyea Jo, Yeon Jeong Kim, Ji Eun Baek, Eun-Soo Kwon, Kwang-Pyo Lee, Siyoung Yang, Ki-Sun Kwon, Dong-Uk Kim, Tae Heung Kang, Yun-Yong Park, Suhwan Chang, Hee Jun Cho, Song Cheol Kim, Sang Seok Koh, Seokho Kim

**Affiliations:** ^1^ Aging Research Institute, Korea Research Institute of Bioscience and Biotechnology, Daejeon, Republic of Korea; ^2^ Department of Biomolecular Science, University of Science and Technology, Daejeon, Republic of Korea; ^3^ Department of Biological Sciences, Dong-A University, Busan, Republic of Korea; ^4^ Department of Immunology, School of Medicine, Konkuk University, Seoul, Republic of Korea; ^5^ Department of Biomedical Sciences and Physiology, University of Ulsan College of Medicine, Seoul, Republic of Korea; ^6^ Departments of Biomedical Sciences and Physiology, Asan Medical Center, University of Ulsan College of Medicine, Seoul, Republic of Korea; ^7^ Immunotherapy Convergence Research Center, Korea Research Institute of Bioscience and Biotechnology, Daejeon, Republic of Korea; ^8^ Department of Surgery, University of Ulsan College of Medicine & Asan Medical Center, Seoul, Republic of Korea

**Keywords:** MDSC, PAUF, pancreatic cancer, tumor microenvironment

## Abstract

Pancreatic cancer is characterized by an immunosuppressive tumor microenvironment (TME) with a profound immune infiltrate populated by a significant number of myeloid-derived suppressor cells (MDSCs). MDSCs have been increasingly recognized for their role in immune evasion and cancer progression as well as their potential as a target for immunotherapy. However, not much is known about the mechanisms regulating their behavior and function in the pancreatic TME. Here we report that pancreatic adenocarcinoma up-regulated factor (PAUF), a soluble protein involved in pancreatic tumorigenesis and metastasis, plays a role as an enhancer of tumor-infiltrating MDSC and its functional activity. We show that PAUF enhanced the accumulation of MDSCs in the spleen and tumor tissues of PAUF-overexpressing tumor cell-injected mice. In addition, PAUF was found to enhance the immunosuppressive function of MDSCs via the TLR4-mediated signaling pathway, which was demonstrated by PAUF-induced increased levels of arginase, nitric oxide (NO), and reactive oxygen species (ROS). The role of PAUF in modulating the functional properties of MDSCs was further demonstrated by the use of a PAUF-neutralizing antibody that caused a decreased number of tumor-infiltrating MDSCs and reduced MDSC immunosuppressive activity. The observations made in mice were confirmed in human pancreatic cancer patient-derived MDSCs, supporting the clinical relevance of our findings. Collectively, we conclude that the PAUF is a powerful and multifunctional promoter of tumor growth through increase and functional activation of MDSCs, suggesting therapeutic potential for targeting PAUF in pancreatic cancers.

## INTRODUCTION

Pancreatic cancer is a highly lethal disease with a 5-year survival rate of < 8% [1]. Despite long-term and ongoing efforts, there has been little success in improving the survival rate; this may be attributed to late diagnoses, its high metastatic ability, and its resistance to therapeutic agents [2]. The limited efficacy of current therapies and the rapid progression of this disease may be explained by abundant tumor-associated stromal content [3]. The pancreatic tumor stroma is comprised of a plethora of cellular and acellular components, including fibroblasts, pancreatic stellate cells, immune cells, endothelial cells, extracellular matrix, and soluble proteins such as cytokines and growth factors [3]. This heterogeneous tumor microenvironment (TME) changes in composition over the course of cancer development, influencing tumor growth, invasion, and sensitivity to therapeutics [3, 4].

One important characteristic of the pancreatic TME is the presence of a massive infiltration of immunosuppressive cells [5–7]. These cells are recruited by tumors as an escape mechanism from immune surveillance and interact with other stromal components to create an immunosuppressive network [8, 9]. One major population of immunosuppressive cells observed in human patients and mouse models of pancreatic cancer is myeloid-derived suppressor cells (MDSCs) [10–13]. MDSCs are a heterogeneous population of immature myeloid cells that are present in most solid tumors [14–16]. They facilitate tumor progression by blocking antitumor immunity through the inhibition of T-cell proliferation, activation, and function, and their critical role in driving immune suppression in the TME makes them an important target for cancer immunotherapy [15, 17, 18].

Immunosuppressive function of MDSCs as well as their accumulation in tumor-surrounding tissues is induced by chronic inflammation that is characteristic of the TME [18, 19]. An inflammatory microenvironment ensues as a result of the secretion of soluble mediators, such as cytokines and chemokines, by tumor and/or stromal cells, with most of the MDSC-inducing soluble factors being directly or indirectly pro-inflammatory [19, 20]. In pancreatic cancer, MDSCs are known to be induced by pro-inflammatory cytokines such as interleukins, granulocyte-macrophage colony-stimulating factor (GM-CSF), interferon gamma (IFN-γ), and tumor necrosis factor (TNF) [20–23]. Although the involvement of these factors in MDSC induction has been established, there still exists a gap in our understanding of the molecular players and mechanisms regulating MDSC behavior and function in the TME.

In our previous study, we identified a novel secreted protein named pancreatic adenocarcinoma up-regulated factor (PAUF) that is highly expressed in pancreatic cancer [24]. This protein plays a role in pancreatic tumor progression and metastasis by acting in an autocrine/paracrine manner [25–29]. PAUF also functions as an endothelial activator that promotes angiogenesis and vascular permeability through the upregulation of C-X-C chemokine receptor 4 (CXCR4) [29] and as an inducer of pro-tumorigenic cytokines [27]. Although its tumorigenic and metastatic role is well documented, little is known about the possible involvement of PAUF in mediating immune evasion during pancreatic cancer's progression.

This study was performed to investigate whether PAUF plays a role in tumor immune evasion through enhancing the properties and functions of MDSCs. We found that MDSC migration and accumulation was significantly increased in PAUF-overexpressing pancreatic cancer cell-injected mice. We also found that the immunosuppressive function of MDSCs is enhanced by PAUF, manifested by the increased production of arginase, nitric oxide (NO), and reactive oxygen species (ROS) through the TLR4-mediated MAPK/ERK pathway. These results were further confirmed by the use of a PAUF-neutralizing antibody in a mouse model of pancreatic cancer and human pancreatic cancer patient-derived MDSCs. These findings have important implications for the development of improved immunotherapy for patients with pancreatic cancer.

## RESULTS

### PAUF contributes to the increasing proportion of MDSCs population in pancreatic cancers

**Figure 1:**
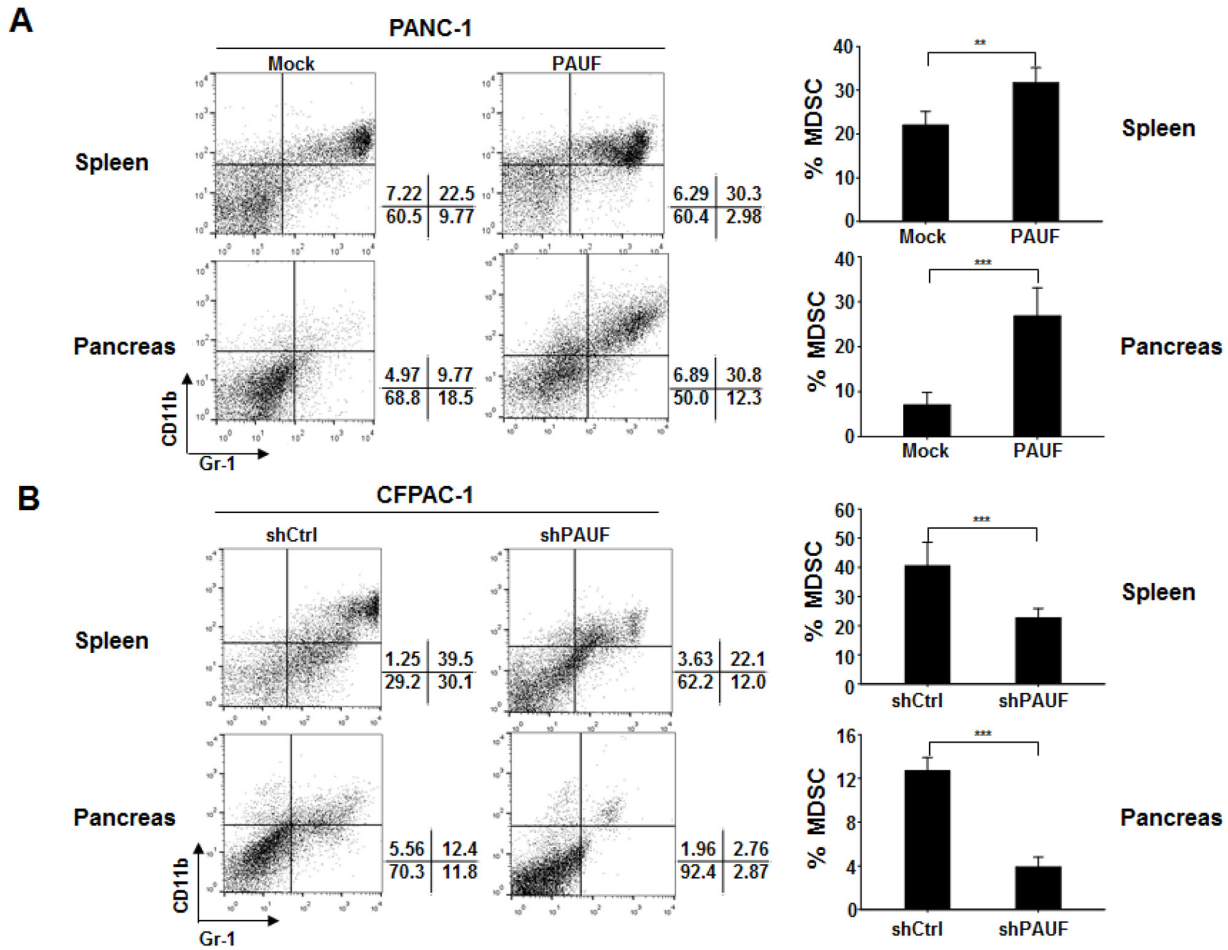
PAUF triggers enhanced MDSC accumulation in pancreatic tumor-bearing mice PANC-1/Mock-Luc or PANC-1/PAUF-Luc **A.** and CFPAC-1/shCtrl or CFPAC-1/shPAUF **B.** cells were orthotopically injected into NOD/SCID mice (*n* = 5). Four weeks after tumor challenge, the proportion of MDSCs in spleen and pancreatic tumor tissues was evaluated by flow cytometry using anti-Gr-1 and anti-CD11b antibodies. Gr-1^+^CD11b^+^ cells were identified as MDSCs. Data represent mean ± S.D. of three independent experiments, and representative images are shown. **, *p* < 0.01; ***, *p* < 0.001.

Previously, it has been shown that PAUF plays an important role in tumor progression and metastasis in pancreatic cancer [24, 25, 27]. However, the role of PAUF in the tumor microenvironment, especially with respect to tumor-immune interplay, remains unknown. To determine whether PAUF affects tumor-neighboring immune cells, particularly MDSCs, which in turn may alter the tumor microenvironment, we orthotopically injected PAUF-overexpressing PANC-1 or control cells with stable luciferase expression (PANC-1/Mock-Luc or PANC-1/PAUF-Luc) into NOD/SCID mice. Four weeks after tumor challenge, we evaluated the number or proportion of MDSCs (Gr-1^+^CD11b^+^) in spleen and/or pancreatic tumor tissues from those mice by flow cytometry. Our *in vivo* bioluminescence imaging analysis revealed that PANC-1/PAUF-Luc xenograft mice developed tumors much larger in volume than PANC-1/Mock-Luc xenograft mice (Supplementary Figure S1A). The proportion of the MDSCs population was significantly increased in spleen and pancreatic tumor tissues from PANC-1/PAUF-Luc cell-injected mice compared to control mice injected with PANC-1/Mock-Luc cells (Figure 1A). Importantly, we detected a significant increase in the absolute number of MDSCs in the tumor tissues from the former group of mice than the latter group (Supplementary Figure S1B). To further confirm these results, we performed the same experiment with CFPAC-1 cells expressing either shRNA against PAUF (CFPAC-1/shPAUF) or control shRNA (CFPAC-1/shCtrl). As shown in Figure 1B, we observed a significant decrease in the proportion of MDSCs in spleen and pancreatic tumor tissues from CFPAC-1/shPAUF cell-injected mice compared to those from CFPAC-1/shCtrl cell-injected mice. These results suggest that PAUF enhances tumor-induced increases of the MDSC population.

### PAUF promotes MDSC recruitment to tumor sites

**Figure 2:**
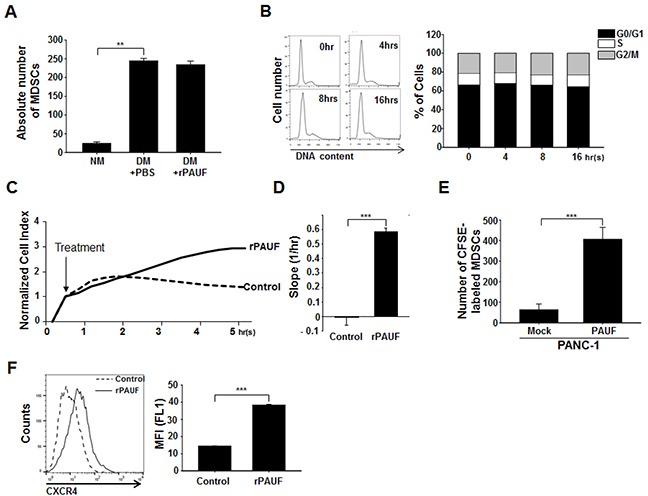
PAUF promotes MDSC migration into tumor tissues **A.** BM cells isolated from C57BL/6 mice were cultured under MDSC-differentiating (GM-CSF- and IL-6-treated) or non-differentiating conditions with cells under differentiating conditions being grown with or without rPAUF (0.5 μg/ml) treatment. After 4-day culture, the absolute number of MDSCs was evaluated by flow cytometry. NM, non-differentiating medium; DM, differentiating medium. **B.** MDSCs treated with rPAUF were analyzed for cell cycle status by propidium iodide (PI) staining and flow cytometry at the indicated time points. The bar graph (right) shows cell cycle distribution percentages at each time point estimated from the DNA content analysis (left). Experiments were performed three times and representative images are shown. **C.** Real-time migration of MDSCs treated or untreated with rPAUF was monitored for 5.5 hours *in vitro* using the xCELLigence system. **D.** The slopes (1/hour) were calculated based on the cell index values shown in (C). **E.** *In vivo* MDSC migration was evaluated by subcutaneous injection of PANC-1/Mock or PANC-1/PAUF cells into both flanks of NOD/SCID mice (*n* = 5), followed by adoptive transfer of CFSE-labeled MDSCs isolated from EL4 tumor-bearing mice after two weeks of tumor challenge, and flow cytometric analysis of tumor-infiltrated MDSCs 24 hours after the adoptive transfer. **F.** CXCR4 expression on EL4 tumor-bearing mice-derived MDSCs treated or untreated with rPAUF for 16 hours was examined by flow cytometry. Data represent mean ± S.D. of three independent experiments, and representative images are shown. MFI, mean fluorescence intensity. **, *p* < 0.01; ***, *p* < 0.001.

To determine whether the PAUF-induced increases in the MDSC population is due to increased MDSC proliferation, migration, or both, we isolated bone marrow (BM) cells from C57BL/6 mice and cultured them under conditions that drive MDSC differentiation in the presence or absence of rPAUF [31]. After a 4 day culture, we determined the absolute number of MDSCs in these cultures by cell counting and flow cytometry. MDSCs grown in the differentiating medium were about 6-fold higher in number compared to those grown in the non-differentiating medium (Figure 2A). However, the absolute number of MDSCs was not affected by rPAUF treatment (Figure 2A), indicating that PAUF may not be involved in promoting MDSC proliferation. To confirm this result, we monitored cell cycle status in MDSCs treated with rPAUF by propidium iodide (PI)-based flow cytometric analysis. As shown in Figure 2B, there was no significant difference in the cell cycle profile among cells treated with rPAUF for durations up to 16 hours. These results led us to investigate whether PAUF is involved in MDSC recruitment to tumor tissues, first by examining *in vitro* MDSC migration using a quantitative real-time monitoring system. As reflected in the cell index as well as the slope, rPAUF-treated MDSCs exhibited significantly increased migration compared to vehicle-treated control cells at 5.5 hours (Figures 2C and 2D). To further confirm this observation *in vivo*, we subcutaneously injected PANC-1/Mock or PANC-1/PAUF cells into both flanks of NOD/SCID mice for 14days tumor challenging, and adoptively transferred CFSE-labeled MDSCs, which were isolated from EL4 tumor-bearing mice. Twenty four hours following the adoptive transfer, we analyzed the number of CFSE-labeled MDSCs infiltrated in tumor tissues by flow cytometric analysis. The number of infiltrated MDSCs significantly increased in PANC-1/PAUF cell-injected mice compared to PANC-1/Mock cell-injected control mice (Figure 2E), demonstrating that PAUF increases migration of MDSC to tumor tissues *in vivo*. Previously, we have shown that PAUF induces expression of CXCR4 on pancreatic cancer cells and dendritic cells [25]. Given that CXCR4 is a key molecule mediating MDSC accumulation in TME [32], we next sought to investigate whether PAUF drives CXCR4 expression on MDSCs. Flow cytometric analysis of CXCR4 expression on MDSCs that were treated or untreated with rPAUF for 16 hours revealed upregulation of CXCR4 on rPAUF-treated MDSCs compared to vehicle-treated control cells (Figure 2F). Taken together, PAUF is correlated with the promotion of MDSC recruitment to tumor tissues, possibly via the upregulation of CXCR4.

### PAUF enhances immunosuppressive functions of MDSCs

**Figure 3:**
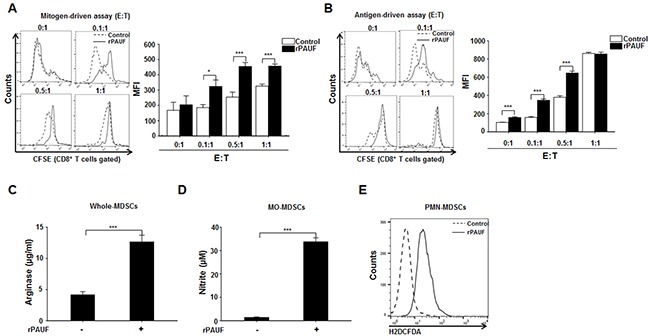
PAUF enhances immunosuppressive function of MDSCs **A.** C57BL/6 mice-derived, CFSE-labeled splenocytes stimulated with anti-CD3 and anti-CD28 antibodies were co-cultured for 3 days with EL4 tumor-bearing mice-derived MDSCs in the presence or absence of rPAUF at indicated effector:target (E:T) ratios. T-cell proliferation was determined based on the mean fluorescence intensity (MFI) values measured in CFSE-labeled Gr-1^+^CD11b^+^ cells by flow cytometry. **B.** The same experiment as (A) was performed except that splenocytes from OT-1 TCR transgenic mice were used. Arginase **C.** NO **D.** and ROS **E.** production by MDSCs treated or untreated with rPAUF was measured as described in Supplementary Materials and Methods. Data represent mean ± S.D. of three independent experiments, and representative results are shown. *, *p* < 0.05; ***, *p* < 0.001.

To determine the involvement of PAUF in the regulation of MDSC function, we examined the inhibitory activity of MDSCs by using mitogen- and antigen-driven T cell proliferation assay after treatment of rPAUF. For a mitogen-driven assay, CFSE-labeled splenocytes from heathy C57BL/6 mice were incubated with anti-CD3 and anti-CD28 antibodies and cultured with rPAUF-treated or untreated MDSCs from EL4 tumor-bearing mice at different effector: target ratios (0:1, 0.1:1, 0.5:1, and 1:1). After 72 hours of co-culturing, the immunosuppressive function of MDSCs was assessed by evaluating the proliferation of CD8^+^ T cells through flow cytometric analysis. rPAUF treatment significantly inhibited CD8^+^ T cell proliferation compared to vehicle treatment (Figure 3A). For an antigen-driven assay, we followed the same procedure except OT-1 TCR transgenic mice-derived splenocytes were used. We similarly observed significant inhibition of OT-1 specific CD8^+^ T cell proliferation by rPAUF treatment (Figure 3B). Inhibition of T cell proliferation by MDSCs is manifested by increased levels of arginase, NO, and ROS, which are known to be produced by Gr-1^+^CD11b^+^ cells, mainly Ly6G^−^Ly6C^high^CD11b^+^ cells (monocytic (MO)-MDSCs), and mainly Ly6G^+^Ly6C^low^CD11b^+^ cells (polymorphonuclear (PMN)-MDSCs) respectively [33, 34]. Given this, we measured the production of arginase, NO, and ROS by the corresponding populations of MDSCs treated or untreated with rPAUF. As expected, we observed a significant increase in arginase and NO production in rPAUF-treated whole MDSCs and MO-MDSCs respectively (Figures 3C and 3D). rPAUF treated PMN-MDSCs also released high levels of ROS in the flow cytometric analysis (Figure 3E). Collectively, our findings suggest that PAUF contributes to the enhanced immunosuppressive function of MDSCs through the increased production of soluble oxidizers.

### PAUF enhances MDSC immunosuppressive activity via TLR4-mediated signaling

**Figure 4:**
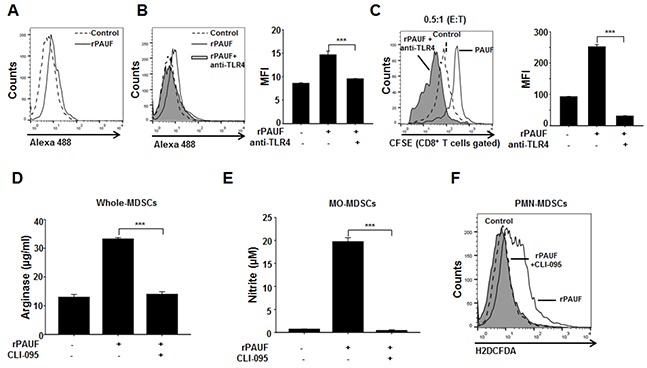
PAUF regulates MDSC immunosuppressive activity via TLR4-mediated signaling **A.** Binding of PAUF to the surface of MDSCs was evaluated by flow cytometric analysis using Alexa 488-labeled PAUF (0.5 μg/ml). Alexa 488 BSA was used as a negative control. Experiments were performed three times, and representative images are shown. **B.** MDSCs were incubated with a TLR4-neutralizing antibody (10 μg/ml) and an isotype control antibody for 1 hour before addition of Alexa 488-labeled PAUF, followed by flow cytometric analysis. MFI, mean fluorescence intensity. **C.** T cell proliferation was evaluated by flow cytometry using rPAUF-treated or untreated MDSCs in the presence or absence of the TLR4-neutralizing antibody. Shown are the results obtained using an effector to target ratio of 0.5:1. Arginase **D.** NO **E.** and ROS **F.** production by MDSCs treated or untreated with rPAUF and/or CLI-095, a TLR4 signaling inhibitor (1 μg/ml), was measured as described in the Supplementary Materials and Methods. Data represent mean ± S.D. of three independent experiments, and representative results are shown. ***, *p* < 0.001.

We have previously shown that PAUF directly binds to TLR4 on the surface of THP-1 and dendritic cells [27, 35]. Given this, we speculated that PAUF may enhance MDSC function through TLR4-mediated signaling in MDSCs. To test this, we first investigated whether PAUF directly binds to the MDSC surface by flow cytometric analysis of EL4 tumor-bearing mice-derived MDSCs treated or untreated with Alexa 488-labeled rPAUF. As expected, rPAUF directly binds to the surface of MDSCs (Figure 4A). To confirm whether PAUF interacts with TLR4 of MDSCs, MDSCs were pre-incubated with a TLR4-neutralizing antibody to block the respective TLR4. We observed the interruption of rPAUF binding on the surface of MDSCs by TLR4-neuralizing antibody (Figure 4B). These results suggest that PAUF interacts with TLR4 on the surface of MDSCs. Furthermore, incubation of rPAUF-treated MDSCs with the TLR4-neutralizing antibody significantly enhanced T cell proliferation at an effector to target ratio of 0.5:1 compared to those untreated with the antibody (Figure 4C). To further explore PAUF-TLR4 interaction mediated signal pathway in MDSCs, we measured arginase, NO, and ROS production using after treatment of CLI-095, a TLR4 signaling inhibitor, with rPAUF. MDSCs double-treated with rPAUF and CLI-095 had a significant reduction in arginase, NO, and ROS production than MDSCs treated with rPAUF only (Figure 4D, 4E, and 4F). These results suggest that PAUF is a ligand of TLR4 and increases the MDSC immunosuppressive function via TLR4-mediated signal pathway.

### PAUF induces MDSCs activation via ERK-mediated AP-1 activation

**Figure 5:**
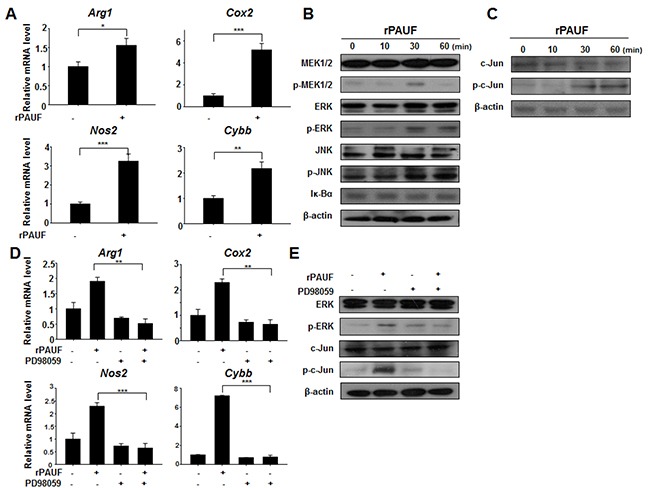
PAUF upregulates immunosuppressive effectors through the ERK signaling cascade in MDSCs **A.** mRNA expression of indicated genes encoding immunosuppressive molecules was analyzed by qRT-PCR in rPAUF-treated or untreated MDSCs. **B.** MDSCs were stimulated with rPAUF for the indicated time periods and lysed for western blotting with antibodies against MEK1/2, phospho (p)-MEK1/2, ERK, p-ERK, JNK, p-JNK, and Iκ-Bα. β-actin was used as a loading control. **C.** The same experiment as (B) was performed and western blotting was carried out with antibodies against c-Jun and p-c-Jun. **D.** mRNA expression of indicated genes encoding immunosuppressive molecules was analyzed by qRT-PCR in rPAUF-treated or untreated MDSCs in the presence or absence of the ERK inhibitor PD98059 (20 μM). **E.** Lysates of rPAUF-treated or untreated MDSCs in the presence or absence of PD98059 (20 μM) for 2 hours were subjected to western blotting with antibodies against ERK p-ERK, c-Jun, and p-c-Jun. Data represent mean ± S.D. of three independent experiments, and representative results are shown. *, *p* < 0.05; **, *p* < 0.01; ***, *p* < 0.001.

To elucidate the molecular mechanisms responsible for PAUF-induced MDSC activation, we examined the expression of genes encoding some of those molecules, including *Arg1*, Cyclooxygenase2 (*Cox2*), inducible nitric oxide synthase 2 (*Nos2*), and cytochrome b-245, beta polypeptide (*Cybb*), by qRT-PCR analysis in rPAUF-treated or untreated MDSCs. As shown in Figure 5A, we observed a significant increase in mRNA levels of these genes in rPAUF-treated MDSCs compared to untreated cells, suggesting that rPAUF regulates transcriptional expression of these immunosuppressive factors. Because the MDSC-derived factors encoded by these genes are induced by AP-1 transcriptional factors through the MAP kinase signaling pathway [36–39], we evaluated whether PAUF induces activation of the MAP kinase signaling pathway in MDSCs. rPAUF treatment of MDSCs increased the phosphorylation of MEK1/2, ERK, and JNK (Figure 5B). In addition, the phosphorylation of these kinases led to activation and translocation of c-Jun, a part of the AP-1 transcription factor (Figure 5C). These results indicate that rPAUF induces AP-1 activation through the ERK signaling pathway. To determine whether PAUF is dependent on the ERK signaling cascade in inducing upregulation of the aforementioned immunosuppressive factors, we examined their mRNA expression in rPAUF-treated or untreated MDSCs in the presence or absence of PD98059, an ERK inhibitor. As expected, PD98059 treatment resulted in the downregulation of these factors at the transcript level (Figure 5D) and a reduction in phosphorylation of c-Jun in rPAUF-treated MDSCs (Figure 5E) compared to PD98059 untreated cells. Taken together, these results indicate that PAUF induces upregulation of immunosuppressive effectors through MAP kinase-dependent AP-1 activation in MDSCs.

### PMAb83, a PAUF-neutralizing antibody, attenuates PAUF-enhanced migration and immunosuppressive activity of MDSCs

**Figure 6:**
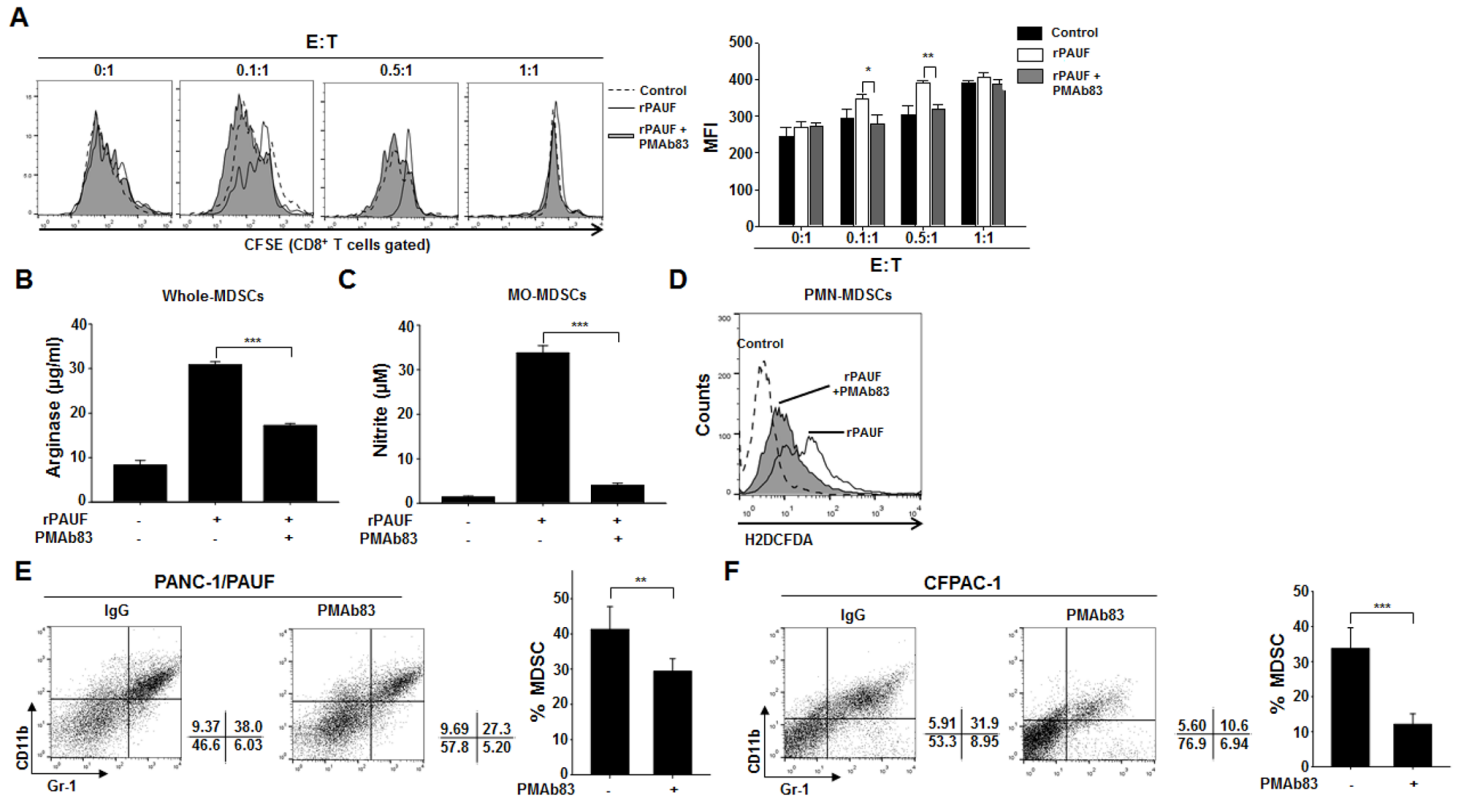
PMAb83 inhibits MDSC accumulation and function *in vitro* and *in vivo* The immunosuppressive function of rPAUF-treated MDSCs was examined in the presence or absence of PMAb83 (15 μg/ml) through a T cell proliferation assay **A.** and analysis of arginase **B.** NO **C.** and ROS **D.** production. *In vivo* accumulation of MDSCs in tumor tissues were evaluated in NOD/SCID mice (*n* = 5) implanted with PANC-1/PAUF **E.** or CFPAC-1 **F.** cells. These mice were administered with control IgG or PMAb83 (10 mg/kg) intraperitoneally twice a week, beginning 5 days after tumor cell injection. After 4 weeks of tumor challenge, they were analyzed for MDSC migration by flow cytometric analysis using anti-Gr-1 and anti-CD11b antibodies. Data represent mean ± S.D. of three independent experiments, and representative results are shown. *, *p* < 0.05; **, *p* < 0.01; ***, *p* < 0.001. MFI, mean fluorescence intensity.

In this study, we showed that PAUF could enhance the immunosuppressive properties of MDSC. Previously, PMAb83, a human monoclonal antibody against PAUF, was a novel targeted therapeutic intervention for pancreatic cancer treatment [30]. To ascertain the influence of neutralized PAUF on MDSCs, we utilized PMAb83 for evaluating the function and migration of MDSCs. We first performed a T cell proliferation assay using PAUF-treated or untreated MDSCs in the presence or absence of PMAb83. We observed a significant increase in T cell proliferation at effector to target ratios of 0.1:1 and 0.5:1 in the group co-cultured with rPAUF- and PMAb83-treated MDSCs compared to rPAUF only-treated cells (Figure 6A). We also investigated arginase, NO, and ROS production under the same experimental conditions and found that the levels of these factors significantly decreased in PAUF-stimulated MDSCs with PMAb83 (Figure 6B, 6C, and 6D). We then examined therapeutic potential of the PMAb83 antibody utilizing orthotopic pancreatic tumor xenograft models. We implanted PANC-1/PAUF cells into pancreas of NOD/SCID mice (n=5) and administered PMAb83 (10mg/kg) twice a week beginning 5 days after tumor cell injection. We found that the population of MDSCs in tumor tissues decreased in PMAb83-administered mice compared to control IgG-injected mice at 4 weeks post-implantation (Figure 6E). Furthermore, we found that the number of MDSCs in tumor tissues from CFPAC-1 cells, expressing the high levels of PAUF, injected mice was significantly lower after administration of PMAb83 to tumor bearing mice (Figure 6F). These results demonstrate that PMAb83 effectively disrupts MDSC accumulation and function *in vitro* and *in vivo*.

### PAUF acts in human MDSCs

**Figure 7:**
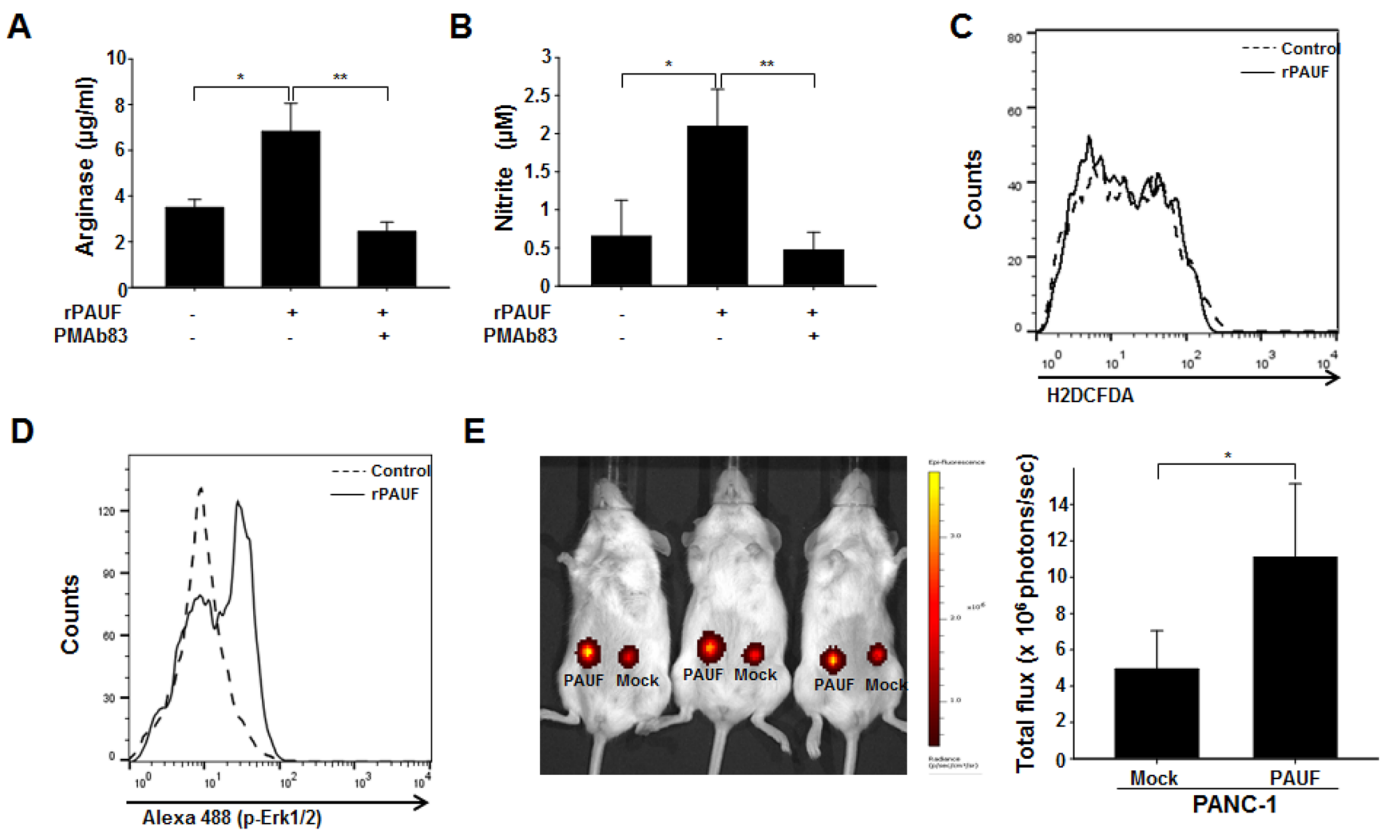
PAUF exerts its role in human MDSCs The PAUF-mediated immunosuppressive function of human MDSCs was evaluated by measuring arginase **A.** NO **B.** and ROS **C.** production by rPAUF-treated or untreated MDSCs in the presence or absence of PMAb83 (15 μg/ml). **D.** Phosphorylation of ERK by PAUF, detected by flow cytometry, was also observed in rPAUF-treated human MDSCs. **E.** *In vivo* MDSC migration was evaluated by subcutaneous injection of PANC-1/Mock or PANC-1/PAUF cells into both flanks of NOD/SCID mice (*n* = 5), followed by adoptive transfer of DiR-labeled MDSCs isolated from human PBMCs after two weeks of tumor challenge, and bioluminescent imaging of tumor-infiltrated MDSCs 24 hours after the adoptive transfer. Data represent mean ± S.D. of three independent experiments, and representative results are shown. *, *p* < 0.05; **, *p* < 0.01.

To test whether the observations are reproducible in human MDSCs, we isolated cells that were double-positive for CD11b and CD33, which are well-known markers of MDSCs [34], from human PBMCs derived from pancreatic cancer patients. We measured arginase, NO, and ROS production by rPAUF-treated or untreated MDSCs in the presence or absence of PMAb83. We observed a significant increase in arginase and NO production by rPAUF-treated MDSCs compared to non-treated cells, which was reversed by PMAb83 (Figure 7A and 7B). Of note is that there was little difference in ROS production between by rPAUF-treated and untreated cells (Figure 7C), which is inconsistent with the results obtained in mice. We also found that PAUF induces phosphorylation of ERK, which was determined by flow cytometry, in human MDSCs as it was in mouse cells (Figure 7D). Consistent with the results from the mouse MDSC migration assay, human MDSC recruitment to tumor tissues significantly increased as well, as demonstrated by a higher number of DiR-labeled human MDSCs infiltrated into tumors in NOD/SCID mice subcutaneously injected with PANC-1/PAUF cells compared to those injected with PANC-1/Mock cells (Figure 7E). All together, these results suggest that PAUF similarly enhances the functional role of human MDSCs.

## DISCUSSION

**Figure 8:**
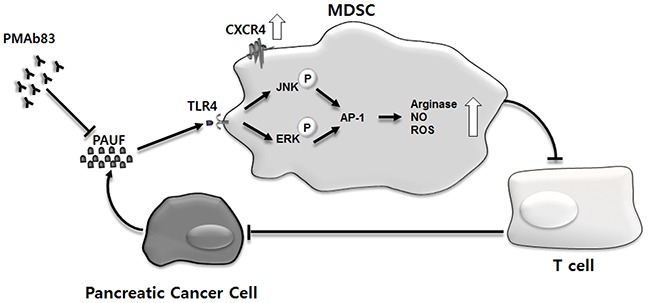
Schematic representation of possible mechanisms of action of PAUF on cells composing the pancreatic TME Pancreatic cancer cells, over the course of tumor development, secrete PAUF, which recruits MDSCs to tumor sites. PAUF plays its role in promoting MDSC migration, in part, via the upregulation of CXCR4. The accumulated MDSCs exert their immunosuppressive function in the TME through TLR4-mediated signaling where PAUF binds to TLR4, phosphorylates ERK and JNK, and activates AP-1. The enhanced immunosuppressive activity of MDSCs is manifested by increased production of arginase, NO, and ROS, which inhibit T cell proliferation, and in turn, impedes antitumor immunity. The MDSC-mediated action of PAUF, which is inhibited by the PAUF-neutralizing antibody PMAb83, contributes to the generation of the uniquely immunosuppressive TME in pancreatic cancer.

Tumor-stroma interactions have emerged as critical players in pancreatic cancer progression, invasion, and metastasis [40–43]. Immune cells in the tumor stroma in particular are increasingly recognized for their role in shaping TME through crosstalk with tumor cells and other stromal components [44–46]. MDSCs are a major component of the pancreatic cancer-associated immunosuppressive microenvironment and their expansion and accumulation is well known to be associated with tumor immune evasion [11, 47, 48]. However, the molecular mechanisms underlying MDSC activation, expansion, and migration are not completely understood in pancreatic cancer. The present study provides strong evidence suggesting that PAUF, a secretory protein with a role in pancreatic cancer progression and metastasis [24, 27], is an important molecular factor involved in the accumulation and function of MDSCs in the TME. We provides a schematic summary of possible PAUF-mediated mechanisms involved in the regulation of MDSCs in the pancreatic TME (Figure 8).

In this study, we observed an increase and a reduction in the number of MDSCs in spleen and tumor tissues from mice injected with PAUF-overexpressing and shPAUF-expressing pancreatic cancer cells, respectively. These results clearly demonstrate the association between PAUF expression and MDSC frequency, and support the role of PAUF in inducing MDSC accumulation similar to that shown for S100A8 and S100A9 in different cellular/cancer contexts [49, 50]. Recent studies have reported an association between the cellular profile and functional status of MDSC subpopulations (such as MO-MDSCs and PMN-MDSCs) and disease state [11, 13]. For example, Khaled *et al*. showed in their study [11] that there is a significant increase in circulating and tumor-infiltrating PMN-, but not MO-MDSCs in pancreatic cancer patients, with arginase 1 expression detected only in circulating PMN-MDSCs. The study conducted by Stromnes and colleagues [13] demonstrated that selected depletion of PMN-MDSCs can unmask an endogenous T cell response in a mouse model of pancreatic ductal adenocarcinoma. Given these, it is tempting to investigate whether PAUF induces the accumulation of (a) specific subpopulation(s) of MDSCs and hence modulates immune responses and influences disease phenotype.

Of note is that PAUF-induced accumulation of MDSCs was not due to increased proliferation, but rather increased trafficking of MDSCs to tumor sites. This was not the case in a previous study where PAUF was found to induce proliferation of pancreatic cancer cells through the upregulation and stabilization of beta-catenin [27]. It appears that there exist distinct factors involved in MDSC proliferation, migration, and activation, and that PAUF is not a component of the MDSC proliferation pathway. The process of MDSC migration is known to be driven by chemokine-chemokine receptor interactions [51], and indeed, our study showed the PAUF-induced upregulation of CXCR4, a chemokine receptor with a key role in the tumor cell-microenvironment crosstalk [52], on MDSCs. CXCR4 expression has been reported to be affected by various signaling molecules [53]. Among other factors, IL-10, reported to upregulate CXCR4 expression in human primary monocytes-macrophages [54], was produced via AP-1 activation [55]. PAUF treatment significantly increased the secretion of IL-10 via AP-1 activation in MDSCs (Supplementary Figure S2A). These results are consistent with those of previous research reporting the induction of CXCR4 by PAUF and a concomitant change in the motility of pancreatic cells [25]. In addition, the importance of CXC-CXCR signaling in the regulation of MDSC mobilization has been demonstrated in many types of cancer, including rhabdomyosarcoma [56], ovarian cancer [32], gastric cancer [57], and colitis-associated cancer [58]. The study by Ding *et al*. [57], for example, indicated that the CXCR5-CXCL13 axis is essential for the migration of CD40-positive MDSCs in gastric cancer and may be a potential target for novel therapeutic development. Given that CXCL12 functions to trigger chemotactic migration in a wide range of CXCR4-expressing cells [59–61], it would be worthwhile to investigate if the CXCR4-CXCL12 axis is also important for the mobilization of MDSCs and subsequent tumor progression through the induction of an immunosuppressive microenvironment.

Our previous studies showed that PAUF binds to TLR2 and TLR4 in THP-1 cells [27] and TLR4 in dendritic cells [35]. Consistent with these observations, PAUF was found to act as a ligand for TLR4 in MDSCs. Importantly, PAUF-mediated MDSC immunosuppressive activity was dependent on the TLR4 signaling cascade, suggesting the significance of the PAUF-TLR4 interaction in MDSC function. In addition, the current study identified MAP kinase-dependent AP-1 activation as a molecular event underlying the PAUF-mediated activation of MDSCs, which was evidenced by the induction of genes encoding AP-1-regulated immunosuppressive factors such as *Arg1, Cox2, Nos2, and Cybb* by PAUF. Collectively, this study is, to our knowledge, the first to demonstrate the role of PAUF in MDSC activation and immunosuppressive function. Further evidence supporting this role of PAUF was obtained by using the PAUF-neutralizing antibody PMAb83, which caused significant induction of T cell proliferation, a reduction in arginase, NO, and ROS levels, and a decreased accumulation of MDSCs in tumor tissues in mice. In concordance with these results, PAUF-induced increases in the production of arginase and NO and ERK activation as well as the decreased production of arginase and NO by PMAb83 were observed in human pancreatic cancer patient-derived MDSCs, which strengthens the clinical relevance of our findings. One thing to note is that ROS levels remained unchanged upon PMAb83 treatment in human patient-derived MDSCs, which warrants further investigation.

Taken together, our study showed that PAUF contributes to the failure of T cell immunosurveillance and pancreatic tumor immune escape through its role in MDSC migration and activation, and provides support for the possibility of delaying pancreatic cancer progression by disrupting tumor stromal components. Currently, a variety of immunotherapeutic strategies are being developed to target MDSCs with the aim to modulate tumor immunity. Further mechanistic studies on PAUF-mediated MDSC function in pancreatic cancer, along with the findings of this study, may be translated into more effective and specific interventions.

## MATERIALS AND METHODS

### Animals and tumor models

Animals were housed in a pathogen-free animal facility at Korea Research Institute of Bioscience and Biotechnology (KRIBB). Six- to eight-week-old littermate mice were used in all experiments, in accordance with the protocols approved by the KRIBB Animal Care and Use Committee.

### Cell culture

Authenticated human and murine cell lines, including PANC-1, CFPAC-1, and EL4, were obtained from the American Type Culture Collection (ATCC, Manassas, VA, USA). All cell lines were maintained at 37°C in a humidified atmosphere containing 5% CO_2_. Generation of PAUF-overexpressing (PANC-1/PAUF) or -knockdown (CFPAC-1/shPAUF) cell lines and their respective controls is described in a previous report [25].

### *In vivo* migration assay

PANC-1/Mock or PANC-1/PAUF cells were subcutaneously injected into both flanks of NOD.CB17-Prkdc^scid^ (NOD/SCID) mice. After two weeks post-injection, these mice were adoptively transferred with 1 × 10^6^ CFSE-labeled MDSCs obtained from EL4 tumor-bearing mice or 2 × 10^5^ DiR-labeled MDSCs isolated from human peripheral blood mononuclear cells (PBMCs). Twenty-four hours following the adoptive transfer, migration of CFSE-labeled MDSCs and DiR-labeled MDSCs were analyzed on FACSCalibur (BD Biosciences, Franklin Lakes, NJ, USA) using FlowJo software (FlowJo, Ashland, OR, USA) and the IVIS Lumina Imaging System (Xenogen, Alameda, CA, USA) using Living Image acquisition and analysis software (Xenogen).

### Preparation of rPAUF, and PMAb83

rPAUF, and PMAb83 (a monoclonal antibody against human PAUF) were generated as previously described [24, 30]. rPAUF treatment was performed at a concentration of 0.5 μg/ml. PMAb83 was used at a concentration of 15 μg/ml in *in vitro* experiments.

### Statistical analysis

Data were obtained from at least three experiments performed in triplicate and analyzed using Student's *t*-test. In calculating two-tailed significance levels for equality of means, equal variances were assumed for the two populations. Results were considered significant when *p*-values were < 0.05.

Additional details regarding the materials and methods are provided in the Supplementary Materials and Methods.

## SUPPLEMENTARY MATERIALS AND METHODS FIGURES


