# Supplementary material for: Pancreatic adenocarcinoma up-regulated factor (PAUF) enhances the accumulation and functional activity of myeloid-derived suppressor cells (MDSCs) in pancreatic cancer
Source: Oncotarget. 2016 Jun 17;7(32):51840–53. doi: 10.18632/oncotarget.10123 (PMC5239518; doi:10.18632/oncotarget.10123)
Supplement: Supplementary file 1 [file oncotarget-07-51840-s001.pdf]

Pancreatic adenocarcinoma up-regulated factor (PAUF) enhances the accumulation and functional activity of myeloid-derived suppressor cells (MDSCs) in pancreatic cancer

SUPPLEMENTARY MATERIALS AND METHODS

Animals and tumor models

C57BL/6 mice were purchased from the KRIBB and used for MDSC generation experiments as well as T cell suppression assays. OT-1 TCR-transgenic mice (C57BL/6-Tg(TcraTcrb)1100Mjb/J) and NOD.CB17-Prkdc^{scid} (NOD/SCID) mice were purchased from the Jackson Laboratory (Bar Harbor, ME, USA) and used for T cell suppression assays and implantable tumor studies, respectively. For xenograft tumor models, 1 x 10^6 cells (PANC-1/PAUF, PANC-1/Mock, CFPAC-1, CFPAC-1/shPAUF, or CFPAC-1/shCtrl) were suspended in PBS and injected orthotopically into pancreas of NOD/SCID mice (n = 5). For *in vivo* bioluminescence imaging and PMAb83 injection experiments, the same procedure was followed using PANC-1/PAUF or PANC-1/Mock cells stably transduced with a lentiviral vector containing the firefly luciferase gene (PANC-1/PAUF-Luc or PANC-1/Mock-Luc; LentiM1.4-Luci, Macrogen, Seoul, Korea) and PANC-1/PAUF or CFPAC-1 cells, respectively. Bioluminescent images were obtained using IVIS Lumina Imaging System (Xenogen, Alameda, CA, USA) and analyzed with Living Image acquisition and analysis software (Xenogen). Antibody injection was performed intraperitoneally using control IgG or PMAb83 at a dosage of 10 mg/kg twice a week for 4 weeks beginning 5 days after tumor cell injection. To study MDSC migration to tumor tissues, 1 x 10^6 cells (PANC-1/PAUF or PANC-1/Mock) in PBS were subcutaneously injected into both flanks of NOD/SCID mice (n = 5).

Cell culture and reagents

The PANC-1, CFPAC-1, and EL4 cell lines were cultured in the medium recommended by the supplier: DMEM for PANC-1 and EL4, and IMDM for CFPAC-1, each supplemented with 10% FBS, 2 mM l-glutamine, 100 µg/ml streptomycin, 100 IU/ml penicillin, and 1 mM sodium pyruvate. These cell lines were periodically authenticated by monitoring cell morphology, growth rates, and mycoplasma contamination, determined using MycoAlert Mycoplasma Detection Kit (Lonza, Rockland, ME, USA).

Flow cytometric analysis

Immediately upon sacrifice of animals, single cell suspensions were prepared from bone marrow (BM), spleen, and tumor tissues. In brief, BM cells were harvested from tibias and femurs by centrifugation, and spleen and tumor cells were mechanically dissociated and filtered through a 70 µm cell strainer, followed by removal of red blood cells (RBCs) with ACK Lysing Buffer (Lonza) and cells counting. Cells were then washed once with PBS (Welgene, Gyeongsan, Korea) and stained in PBS containing 0.5% FBS with the following anti-mouse antibodies and their respective isotype controls: fluorescein isothiocyanate (FITC)-conjugated CD11b and CXCR4, phycoerythrin (PE)-conjugated Ly6G and Gr-1, allophycoyanin (APC)-conjugated Ly6C and CD11b from BD Biosciences (Franklin Lakes, NJ, USA), Alexa Fluor 488-conjugated phospho-p44/42 (ERK1/2) MAPK (Thr202/Tyr204), Alexa Fluor 674-conjugated phospho-Stat3 (Tyr705) from Cell Signaling Technology (Danvers, MA, USA).

Generation of MDSCs from bone marrow progenitors

Tibias and femurs were removed from C57BL/6 mice using sterile techniques and BM cells were flushed with syringe, followed by depletion of RBCs using ACK Lysing Buffer (Lonza). To induce differentiation of MDSCs from BMs *in vitro*, 5 x 10^5 cells were plated in 6-well plates in 3 ml of RPMI 1640 supplemented with 10% FBS, 40 ng/ml GM-CSF, and 40 ng/ml IL-6, as described in [1]. Cells were maintained at 37°C in a humidified atmosphere containing 5% CO_2 for 4 days. For cell cycle analysis, MDSCs were seeded at 5 x 10^5 cells per well in 6-welll plates and treated with rPAUF (0.5 µg/ml) for 0-16 hour(s). Cell cycle profile was determined by propidium iodide (PI) staining of nuclear DNA using CycleTEST PLUS kit (BD Biosciences), followed by flow cytometry on FACSCalibur (BD Biosciences). Data were analyzed using FlowJo software (FlowJo, Ashland, OR, USA).

MDSC isolation and *in vitro* characterization

MDSCs were isolated from tumor tissues of EL4 tumor-bearing mice. For *in vivo* experiments, freshly isolated cells were purified by immunomagnetic separation using biotinylated anti-CD11b and anti-Gr-1 antibodies and streptavidin-conjugated MicroBeads with MidiMACS

separators (Miltenyi Biotec, Bergisch Gladbach, Germany) following the manufacturer's instructions. *In vitro* experiments were carried out using the whole MDSC population (Gr-1$^+$CD11b$^+$ cells), monocytic (MO) MDSCs (Ly6G$^-$Ly6C^{high}CD11b$^+$ cells), and/or polymorphonuclear (PMN) MDSCs (Ly6G$^+$Ly6C^{low}CD11b$^+$ cells), which were isolated using the Myeloid-Derived Suppressor Cell Isolation Kit (Miltenyi Biotec) and purified using streptavidin-conjugated MicroBeads with MidiMACS separators (Miltenyi Biotec). Cell viability was assessed by trypan blue exclusion, and cell purity, which was > 95%, was determined by flow cytometry.

CXCR4 expression on MDSCs was examined by flow cytometry after 16-hour treatment with rPAUF (0.5 µg/ml). For inhibition of ERK signaling, MDSCs were treated with PD98059 (20 µM) or vehicle (dimethylsulfoxide) and incubated for 2 hours before rPAUF (0.5 µg/ml) treatment for 30 minutes for qRT-PCR analysis or for 0-60 minute(s) for western blotting analysis. To examine PAUF-TLR4 interaction, MDSCs were treated for 1 hour with TLR4-neutralizing antibody at a concentration of 10 µg/ml or CLI-095 from InvivoGen (San Diego, CA, USA) at a concentration of 1 µg/ml before addition of rPAUF (0.5 µg/ml) for 30 minutes.

Human blood samples were obtained from patients with pancreatic ductal adenocarcinoma (PDAC) according to the protocols approved by the Institutional Review Board (IRB) of the Asan Medical Center in Korea. All patients provided informed consent. Peripheral blood samples were collected in BD Vacutainer K2 EDTA 5.4mg (BD Diagnostics, Franklin Lakes, NJ, USA), and peripheral blood mononuclear cells (PBMCs) were isolated by Ficoll density centrifugation. These cells were then washed with 0.5% BSA in PBS, and purified using anti-CD33 and anti-CD11b MicroBeads with MiniMACS separators (Miltenyi Biotec) according to the manufacturer's protocol. Cell purity, determined by flow cytometry, was > 95%.

Real-time cell migration analysis

MDSC migration was evaluated using the xCELLigence System (Roche Applied Science, Mannheim, Germany), which allows label-free, real-time monitoring of cell migration. More details of this system are described elsewhere [2]. For a migration assay, the lower and upper chambers were filled with cell-free complete medium and serum-free medium containing none- or rPAUF-treated MDSCs at a density of 3 x 10^4 cells/well, respectively. Cell index (CI) values, which reflect electrical impedance changes caused by cell migration from the upper to the lower chambers, were obtained at 15-minute intervals from the time of plating until the end of the experiment for a total of 5.5 hours. The data obtained were analyzed using the Real-Time Cell Analyzer software (Roche Applied Science).

Quantitative real-time RT-PCR (qRT-PCR)

Total RNA was extracted from MDSCs isolated from BM using the RNeasy Mini Kit (QIAGEN, Valencia, CA, USA) and extracted RNA was quantified by measuring absorbance at 260 nm using an ND-1000 spectrophotometer (NanoDrop Technologies, Wilmington, DE, USA). cDNA was synthesized from 1 µg RNA using the AccuPower CycleScript RT PreMix Kit (Bioneer, Daejeon, Korea). Real-time RT-PCR was conducted on a CFX96 Touch Real-Time PCR Detection System (Bio-Rad, Hercules, CA, USA) using the GoTaq Probe qPCR Master Mix (Promega Corporation, Madison, WI, USA) and the following gene-specific primers: *Arg1*, (forward) 5'-AACACGGCAGTGGCTTTAACCT-3' and (reverse) 5'-GTGATGCCCCAGATGGTTTTC-3'; *Cox2*, (forward) 5'-CCCTGAAGCCGTACACATCA-3' and (reverse) 5'-TGTCACTGTAGAGGGCTTTCAATT-3'; *Nos2*, (forward) 5'-GACCCAGATGCAGGAAAGGAA-3' and (reverse) 5'-GAAACTATGGAGCACAGCCACAT-3'; *Cybb*, (forward) 5'-GACCCAGATGCAGGAAAGGAA-3' and (reverse) 5'-TCATGGTGCACAGCAAA GTGAT-3'. Real-time RT-PCR data were obtained in the form of threshold cycle (Ct) values, and target gene expression was normalized to beta-actin expression. Relative expression levels of target genes (*Arg1, Cox2, Nos2, and Cybb*) were calculated by the comparative Ct ($2^{-\Delta\Delta Ct}$) method as previously described [3, 4].

T cell suppression assay

Freshly isolated splenocytes (5 x 10^6 cells/ml) from OT-1 TCR transgenic mice were depleted of RBCs, labeled with 1 µM 5(6)-carboxyfluorescein N-hydroxysuccinimidyl ester (CFSE, Molecular Probes, Carlsbad, CA, USA) for 10 minutes at 37˚C, and then washed with fresh culture media. These OT-1 splenocytes were specifically stimulated with OVA257-264 peptide (1 µg/ml, AnaSpec, Fremont, CA, USA) or nonspecifically stimulated with anti-CD3 (5 µg/ml) and anti-CD28 (2 µg/ml) antibodies from BD Biosciences. The purified whole MDSC population or NO-/PMN-MDSC subset from the BM was cultured in RPMI 1640 supplemented with 10% FBS for 3 days in U-bottom 96-well plates with 2 x 10^5 CFSE-labeled splenocytes at different ratios of 0:1, 0.1:1, 0.5:1, and 1:1. Cells were analyzed on FACSCalibur (BD Biosciences), and data were processed using FlowJo software (FlowJo) and represented as mean fluorescence intensity (MFI) of CFSE in CD8$^+$ T cells.

Arginase activity assay

Arginase activity assay was performed on Gr-1^+CD11b^+ cells (1×10^6) as described by Bayne *et al*. (2012) [5].

Measurement of NO and ROS production

Nitric oxide production by Gr-1^+CD11b^+ cells was examined in culture supernatant from the T cell suppression assay as previously described [5]. To measure ROS production, MDSCs were treated with 10 μM 2’,7’-dichlorodihydrofluorescein diacetate (H_2DCFDA) in the dark. After 10-minute incubation at 37°C, dichlorofluorescein fluorescence was measured by flow cytometry using FACSCalibur (BD Biosciences).

Western blotting analysis

MDSCs were lysed in RIPA (Radio-Immunoprecipitation Assay) buffer containing protease and phosphatase inhibitor cocktails. The rest of procedure was carried out as described in [6] except that 10% SDS-PAGE gels were used and that incubation with peroxidase-conjugated secondary antibodies was performed for 1 hour at room temperature. Results were visualized by chemiluminescence using WEST SAVE UP (AbFrontier, Seoul, Korea). Antibodies against MEK1/2, p-MEK1/2, ERK, p-ERK, JNK, p-JNK, c-Jun, p-c-Jun, and Ik-Ba were obtained from Cell Signaling Technology. Beta-actin from AbFrontier was used as a loading control.

REFERENCES

1. Marigo I, Bosio E, Solito S, Mesa C, Fernandez A, Dolcetti L, Ugel S, Sonda N, Bicciato S, Falisi E, Calabrese F, Basso G, Zanovello P, Cozzi E, Mandruzzato S and Bronte V. Tumor-induced tolerance and immune suppression depend on the C/EBPbeta transcription factor. Immunity. 2010; 32:790-802.
2. Bird C and Kirstein S. Real-time, label-free monitoring of cellular invasion and migration with the xCELLigence system. Nature Methods. 2009; 6:v-vi.
3. Schmittgen TD and Livak KJ. Analyzing real-time PCR data by the comparative C(T) method. Nat Protoc. 2008; 3:1101-1108.
4. Livak KJ and Schmittgen TD. Analysis of relative gene expression data using real-time quantitative PCR and the 2(-Delta Delta C(T)) Method. Methods. 2001; 25:402-408.
5. Bayne LJ, Beatty GL, Jhala N, Clark CE, Rhim AD, Stanger BZ and Vonderheide RH. Tumor-derived granulocyte-macrophage colony-stimulating factor regulates myeloid inflammation and T cell immunity in pancreatic cancer. Cancer Cell. 2012; 21:822-835.
6. Capietto AH, Kim S, Sanford DE, Linehan DC, Hikida M, Kumosaki T, Novack DV and Faccio R. Down-regulation of PLCgamma2-beta-catenin pathway promotes activation and expansion of myeloid-derived suppressor cells in cancer. J Exp Med. 2013; 210:2257-2271.

SUPPLEMENTARY FIGURES

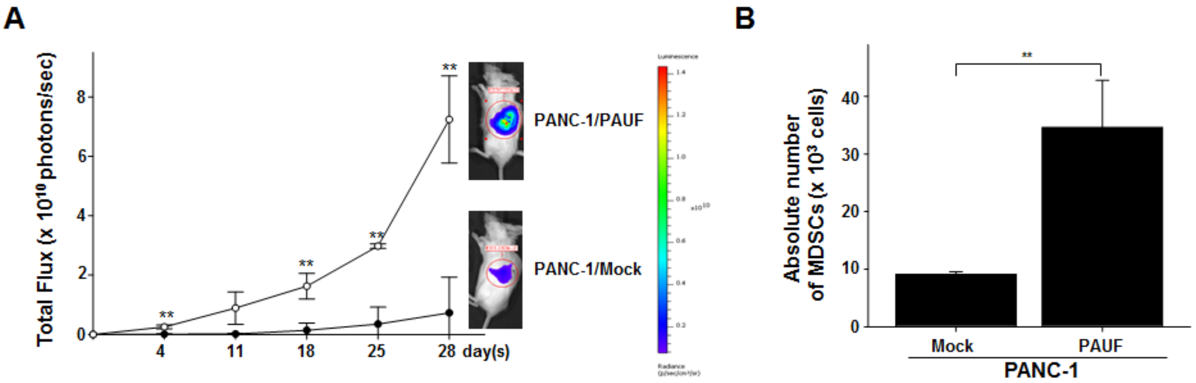

Supplementary Figure S1: PAUF enhances pancreatic tumor progression and MDSC recruitment. 1×10^6 PANC-1/PAUF or PANC-1/Mock cells stably expressing the firefly luciferase gene were orthotopically implanted into pancreas of NOD/SCID mice (n = 5 for each group). **A.** Bioluminescent tumor signals quantified as total flux (photons/sec, mean ± S.D.) were measured *in vivo* on the indicated days and plotted against time course with representative whole body images from each group taken on day 28 shown. **B.** MDSC (Gr-1^+CD11b$^+$) recruitment was analyzed in pancreatic tumor tissues obtained on day 28 post-implantation and quantified as absolute number of cells (mean ± S.D.) using flow cytometry. **, $p < 0.01$.

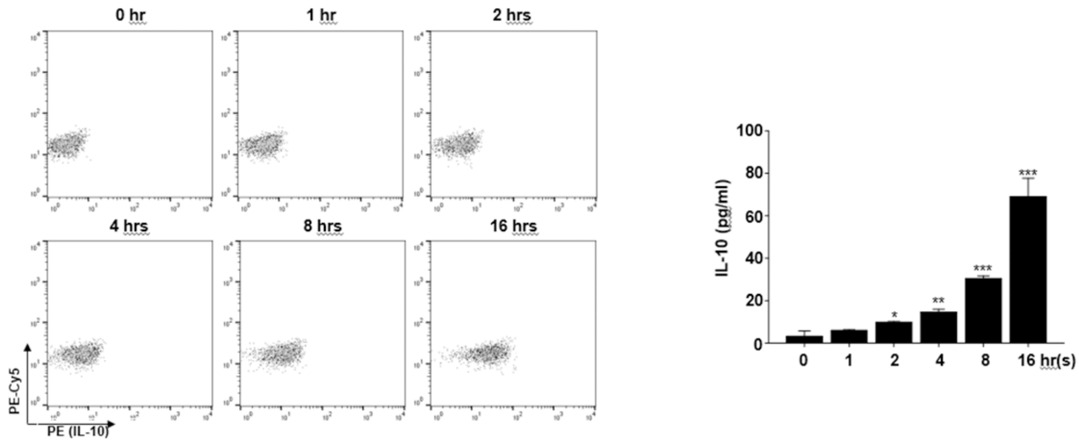

Supplementary Figure S2: Quantitative analysis of IL-10 secretion by PAUF-treated and untreated MDSCs. EL4 tumor-bearing mice-derived MDSCs were cultured in the presence or absence of rPAUF (0.5 μg/ml) for up to 16 hours. Time-course change in IL-10 levels in the culture media was evaluated by flow cytometry using the BD Cytometric. Bead Array (CBA) Mouse Th1/Th2/Th17 Cytokine Kit (BD Biosciences) according to the manufacturer's instructions. Flow cytometry-based quantification of IL-10 levels (left) were represented as bar graph (right). *, $p < 0.05$; **, $p < 0.01$; ***, $p < 0.001$.
